# Genomic and biological characterization of a velogenic Newcastle disease virus isolated from a healthy backyard poultry flock in 2010

**DOI:** 10.1186/1743-422X-9-46

**Published:** 2012-02-16

**Authors:** Muhammad Munir, Muhammad Abbas, Muhammad Tanveer Khan, Siamak Zohari, Mikael Berg

**Affiliations:** 1 Division of Virology, Department of Biomedical Sciences and Veterinary Public Health, Swedish University of Agricultural Sciences (SLU), Ulls väg 2B, 751 89 Uppsala, Sweden; 2 Quality Control Section Veterinary Research Institute (VRI), Lahore, Pakistan; 3 Department of Virology, Immunobiology and Parasitology, National Veterinary Institute (SVA), Ulls väg 2B, 751 89 Uppsala, Sweden

**Keywords:** Newcastle disease, Rural poultry, DNA sequencing, Genome characterization, Pakistan

## Abstract

**Background:**

Newcastle disease virus (NDV) causes severe and economically important disease in poultry around the globe. None of NDV strains in Pakistan have been completely characterized and the role of rural poultry in harbouring NDV is unclear. Since they have a very important role for long-term circulation of the virus, samples were collected from apparently healthy backyard poultry (BYP) flocks. These samples were biologically analyzed using mean death time (MDT) and intracerebral pathogenicity index (ICPI), whereas genotypically characterized by the real-time PCRs coupled with sequencing of the complete genome.

**Findings:**

Despite of being non-pathogenic for BYP, the isolate exhibited MDT of 49.6 h in embryonated chicken eggs and an ICPI value of 1.5. The F gene based real-time PCR was positive, whereas M-gene based was negative due to substantial changes in the probe-binding site. The entire genome of the isolate was found to be 15192 nucleotides long and encodes for six genes with an order of 3'-NP-P-M-F-HN-L-5'. The F protein cleavage site, an indicative of pathogenicity, was ^112^RRQKRF^117^. Complete genome comparison indicated that the RNA dependent RNA polymerase gene was the most and the phosphoprotein was least conserved gene, among all the genes. The isolate showed an Y526Q substitution in the HN protein, which determines neuraminidase receptor binding and fusion activity of NDV. Phylogenetic analysis, based on F and HN genes, classified this isolate into genotype VII, a predominant genotype responsible for ND outbreaks in Asian countries. However, it clustered well apart from other isolates in this genotype to be considered a new subgenotype (VII-f).

**Conclusions:**

These results revealed that this isolate was similar to virulent strains of NDV and was avirulent in BYP either due to resistance of local breeds or due to other factors such as substantial mutations in the HN protein. Furthermore, we have characterized the first isolate of NDV, which could act as domestic reference strain and could help in development and selection of appropriate strain of NDV for vaccine in the country.

## Background

The Newcastle disease virus (NDV) belongs to genus *Avulavirus *within family *Paramyxoviridae*, order *Mononegavirales *[1]. The virus is enveloped with a single-stranded with negative sense RNA genome. The genome is approximately 15 kb in length and follows the "rule of six" which is a pre-requisite for efficient viral replication [2]. The genome (3' to 5') encodes for 6 different proteins, i.e. nucleoprotein (NP), phosphoprotein (P), fusion protein (F), matrix protein (M), hemagglutinin-neuraminidase (HN), and RNA large polymerase (L) protein. The NP, P and M proteins encompass the viral inner surface whereas the L protein constitutes the viral nucleocapsid together with NP and P proteins. The two surface glycoproteins HN and F are responsible for binding to host cell sialic acid receptors and for fusion of the viral envelop to the host cell membrane, respectively [3]. As a property of the family, the NDV carries high protein coding capacity, which is further enhanced by the mechanism called "RNA editing". This mechanism results in generation of V and W proteins with one or two guanines (G) insertion, respectively, during the transcription of P gene mRNA [4].

On the basis of conventional *in vivo *pathogenicity indices for chicken, NDV strains can be divided into pathotypes. The viscerotropic velogenic NDV is highly pathogenic and causes intestinal infection with high mortality, whereas neurotropic velogenic NDV is responsible for symptoms of the respiratory and nervous systems with high mortality. The mesogenic strains are relatively less pathogenic, often with acute respiratory and nervous symptoms but with relatively low mortality. The lentogenic strains of NDV cause mild respiratory tract infections. It is asymptomatic enteric form in which host live longer and it is a privilege to virus for replication and shedding [3]. This difference in pathogenicity is primarily due to differences in the cleavage site within the F protein. This protein is synthesized as a precursor (F_0_) in non-functional state, which then is cleaved by host proteases into two functionally active polypeptides (F_1 _and F_2_). All the mesogenic and velogenic strains of NDV carry an amino acid sequence of ^112^R/K-R-Q-R/K-R-F^117 ^within the F protein whereas lentogenic viruses have ^112 ^G/E-K/R-Q-G/E-R-L^117 ^[5].

On the basis of phylogenetic analysis with the partial hypervariable nucleotide sequences of the F gene, NDV strains have been classified into ten genotypes (I-X). The five genotypes (I, II, III, IV, IX) are considered old (1930-1960) and remaining five genotypes (V, VI, VII, VIII, X) are considered recent (after 1960). However, all have indistinguishable pathogenicity in their hosts. The genotype VI and VII are further divided into seven (VIa-g) and five (VIIa-e) subgenotypes, respectively [6,7]. In parallel, an alternative pattern for NDV classification exists which was initially presented by Aldous et al., [8] while conducting studies on a large number of NDV isolates collected from several countries. According to this criterion, the NDV can be grouped into six distinct genetic lineages (1-6) with several sublineages within them. There are around fifty-five complete genomes of different NDV strains available, which can be divided into three different genome lengths: 15186 nt, 15192 nt and 15198 nt [9-11].

Newcastle disease (ND) is an OIE notifiable disease and notification of any outbreak is mandatory to the OIE [12]. ND is distributed around the globe and is consistently reported from all the continents. In Pakistan, a sporadic form of the disease exists throughout the year, and only a limited number of outbreaks are officially or unofficially reported annually. Despite the extensive and unrestricted use of imported vaccines, NDV still remains the main poultry disease in both commercial and rural chickens of Pakistan [13]. Incompatibility between field and vaccine strains and generation of novel NDV strains explain this failure of vaccine. Moreover, the role of rural poultry in the epizootiology of NDV in the country has always remained a mystery. In order to evaluate the degree of genetic diversity of NDV strains circulating in backyard poultry and to estimate the relationships to that of NDV currently circulating in the region, the complete genome of NDV isolated from healthy backyard poultry flocks was characterized genetically, phylogenetically and biologically.

## Results

### Pathogenicity assessment

Ten out of 12 collected samples from apparently healthy backyard poultry flocks were recovered from specific pathogen free (SPF) embryos and showed heamagglutination (HA) titer. Surprisingly, the isolated virus was found virulent by OIE standard criteria. Chicken/BYP/Pakistan/2010 exhibited mean death time (MDT) of 49.6 h in embryonated chicken eggs. The intracerebral pathogenicity index (ICPI) value of Chicken/BYP/Pakistan/2010 was calculated to be 1.5. These results revealed that Chicken/BYP/Pakistan/2010 was similar to virulent strains of NDV, regarding pathogenicity.

### Nucleic acid detection

The positive samples were screened for the presence of Newcastle disease virus by real-time reverse transcription polymerase chain reaction (rRT-PCR) for the matrix and fusion protein genes. All the ten isolates were found positive with F gene based real-time PCR. However, M gene based real-time PCR failed to detect even a single sample.

### Phylogenetic analysis

**Figure 1:**
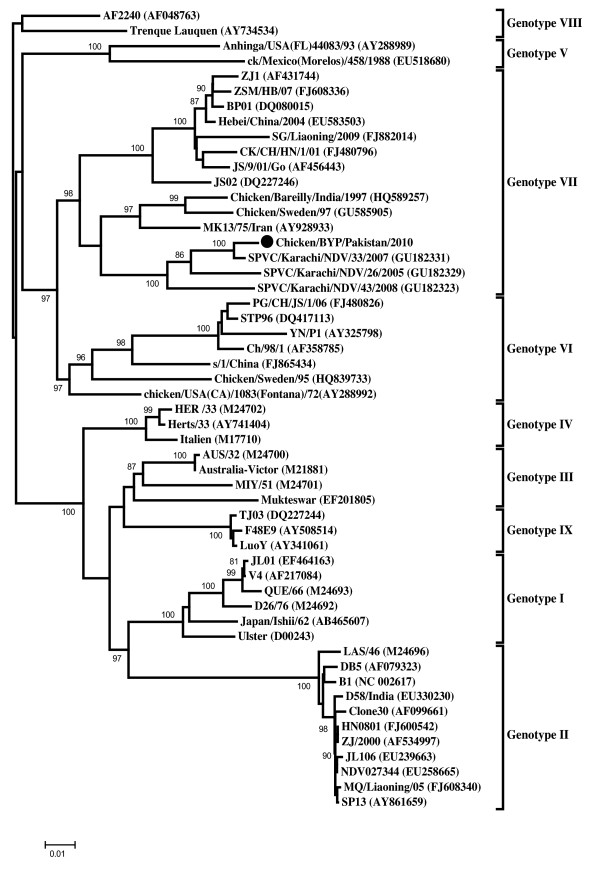
**Phylogenetic tree based on the complete open reading frame of fusion genes of Chicken/BYP/Pakistan/2010 and NDV isolates representing all the genotypes**. The isolate used in this study is marked with black circle (●).

The phylogenetic relationships of Chicken/BYP/Pakistan/2010 with other members of NDV were obtained by comparing the nucleotide sequences of the complete coding region of the F gene representing the IX genotypes. The resulting phylogenetic tree is depicted in Figure 1. From the topology of the Bayesian tree, presented in Figure 1, it was apparent that the NDV isolate under study was placed close to genotype VII. However, Chicken/BYP/Pakistan/2010 showed only 89.0% nucleotide similarity to that of NA-1 (DQ659677), a representative of genotype VII, when the complete genomes of both isolates were compared. Based on the frequency distribution criteria for genotype classification, Chicken/BYP/Pakistan/2010 could therefore be considered as separate genotype/subgenotype (Table 1). The existence of deep rooted branching for the isolate and clustering well apart from the rest of sequences within genotype VII provided substantial evidences that support this isolate as separate genotype/subgenotype. Being a member of genotype VII, Chicken/BYP/Pakistan/2010 clustered to the isolates from India, Iran and Sweden along with other Pakistani isolates sequenced from 2005-08. In general, NDV isolates from other Asian countries such as China, Japan and Taiwan constitute genotype VII.

**Table 1:** Pairwise sequence comparison of the F gene sequences belonging to all the subgenotypes within genotype VII

	p-distance (%)					
**Group**	**VIIa**	**VIIb**	**VIIc**	**VIId**	**VIIe**	**VIIf**

Subgenotype VIIa	0					

Subgenotype VIIb	5.3	0				

Subgenotype VIIc	5.2	7.5	0			

Subgenotype VIId	4.7	7.6	5.5	0		

Subgenotype VIIe	6.9	6.6	10.0	8.9	0	

Subgenotype VIIf	8.9	5.9	10.0	10.2	9.3	0

Using MEGA4 programme the data was generated and the values indicate% nucleotide sequence distances

**Figure 2:**
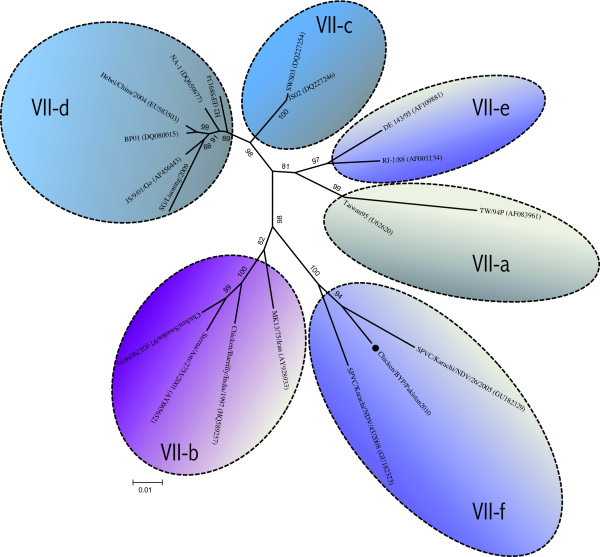
**Phylogenetic tree based on the complete open reading frame of fusion genes of Chicken/BYP/Pakistan/2010 and NDV isolates representing all the subgenotypes within genotype VII**. The isolate used in this study is marked with black circle (●).

The genotype VII can further be divided into five subgenotypes (VIIa-e) as presented in Figure 2, and Chicken/BYP/Pakistan/2010 clustered specifically close to subgenotype b (VII-b). However, it showed significant differences to rest of the subgenotypes and it clustered apart. Hence, tentatively could be considered as a new subgenotype (VII-f). Notably, the Pakistani isolates from previous study also clustered with Chicken/BYP/Pakistan/2010 within VII-f.

**Figure 3:**
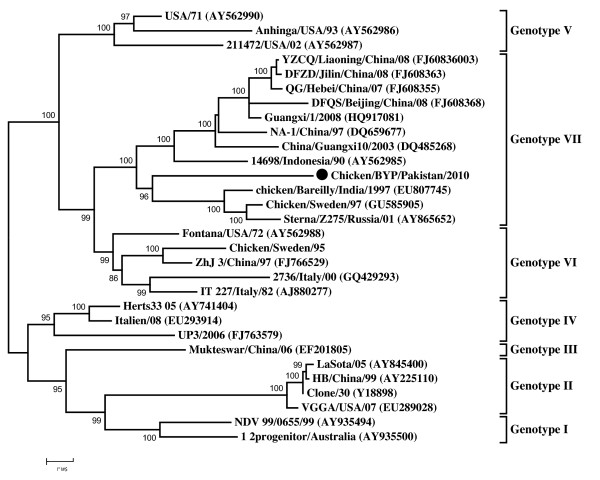
**Phylogenetic tree based on the complete open reading frame of hemagglutinin-neuraminidase genes of Chicken/BYP/Pakistan/2010 and NDV isolates representing all the genotypes**. The isolate used in this study is marked with black circle (●).

It has been speculated that HN gene can differentiate the genotypes of NDV clearly and may true predict the pathogenicity of the isolates because the length of HN protein varies and cleavage site is not the sole criteria for pathogenicity [11,14]. Therefore, the phylogenetic analysis was conducted using complete coding region of the HN gene. In general, the same topology of the tree was observed as seen with the Bayesian tree of F gene analysis. The Chicken/BYP/Pakistan/2010 clustered together in genotype VII as expected, in relation to its HN protein length of 571 aa (Figure 3).

### Genomic and non-coding sequence analysis

ND viruses can be divided into groups based on genome lengths, with earlier lineages (I-IV) having a length of 15186 nt, recent lineages (V-VII) a length of 15192 nt due to insertion of 6 nt into the 5' non-coding region of the NP gene, and the class 1 APMV-1 a length of 15198 nt with the insertion of 12 nt into the coding region of P gene. The length of Chicken/BYP/Pakistan/2010 was 15192 nt (GenBank accession number JN682210) and is thus follow the "rule of six", a feature that has been found essential for the effective viral replication. As typical among paramyxoviruses, the genome was organized as NP-P/V/W-M-F-HN-L from 3' to 5' end of the genome. The protein coding capacity of the genome was estimated to be 90.4% and the GC content 46%. Among the full-length sequences of NDV available in the GenBank, Chicken/BYP/Pakistan/2010 showed highest nucleotide sequence similarity with Sterna/Astr/2755/2001 (GenBank accession number AY865652). This virus was isolated from a little tern (*Sterna albifrons Pallas*) in the Volga river delta in Russia.

The genome of APMV-1 starts with a stretch of sequences known as leader at the 3'end, and ends at the trailer sequence at the 5'end. The 3'-leader sequence serves as a promoter from where transcription of mRNA starts and continues through a mechanism commonly known as "start-stop-restart". Each gene of the APMV-1 starts with a relatively conserved sequence of gene start (GS) and ends at a sequence of gene end [6]. The open reading frame (ORF) of each gene overhangs with 3'and 5' untranslated regions (UTRs) on their respective ends. Between GE of one gene and GS of the next, a conserved sequence exists known as intergenic sequence (IGS). All these features for Chicken/BYP/Pakistan/2010 are summarized in Table 2. The GS and GE sequences were found to be conserved among the genes. An identical GS (ACG_3_TAGA_2_) was observed for the first five genes (NP, P, M, F, and HN), whereas one nucleotide difference (ACG_3_TAG_2_A) was seen in the GS of the last gene (L). The GE sequence in the NP, M, HN and L genes (T_2_AGA_6_) was different from that in the P and F genes (T_2_A_2_GA_6_). This feature is common among NDV strains of genotype VII [11].

**Table 2:** Genome characteristics of Chicken/BYP/Pakistan/2010

Genes	Genome characteristics					**Intergenic region [**6**]**	**Gene length [**6**]**	**Protein length [**15**]^b^**
				
	Gene start (from-to)	**3**'**UTR**	Coding sequence (from-to)^a^	**5**'**UTR**	Gene end (from-to)			
**NP**	56-65	66	122-1591	216	1798-1807	2	1752	489

**P**	1810-1819	83	1893-3080	180	3250-3260	1	1451	395

**V**	-	-	-	-	-	-	-	239

**W**	-	-	-	-	-	-	-	227

**M**	3262-3271	34	3296-4390	112	4493-4502	1	1241	364

**F**	4504-4513	46	4550-6211	84	6285-6295	31	1791	553

**HN**	6327-6336	91	6418-8133	195	8319-8328	47	2002	571

**L**	8376-8385	11	8387-15001	77	15069-15078	-	6702	2204

^a^including stop codon, ^b^without stop codon

The 3'-terminus sequence (1-19 nt) and 5'-terminus sequence (15173-15192 nt) of Chicken/BYP/Pakistan/2010 was uncertain because the primers were designed based on the consensus generated by the alignment of the 52 complete genomes. However, the comparison of these sequences revealed that both termini are conserved especially the first 12 nt and last 8 nt in the 3'and 5'ends of the genome, respectively.

### Features of the coding region

#### NP gene analysis

The NP gene of Chicken/BYP/Pakistan/2010 showed a highest nucleotide and amino acid similarity to that of NA-1 (DQ659677) and IT-227/82 (AJ880277), respectively (Table 3) when compared to representatives of each genotype. The other features of all the genes such as gene length, ORF length, GS, GE and predicted molecular weight are summarized in Table 2. All APMV-1 carries a stretch of 15 amino-acids (N'-FX_4_YX_3_ΦSΦAMG-C', where × is any amino acid and Φ is any aromatic amino acid), in the middle of NP ORF, which is responsible for the N-N self assembly in the process of RNA binding and this sequence in Chicken/BYP/Pakistan/2010 was ^322^FAPAEYAQLYSFAMG^336^.

**Table 3:** Percentage similarity between the nucleic acid and amino acid sequences of each of the open reading frames and the complete genome of Chicken/BYP/Pakistan/2010 and a representative sequence from each genotype

Strain (accession no)	Ulster (AY562991)		LaSota (AY845400)		Mukteswar (EF201805)		Hert/33 (AY7411404)		Largo/71 (AY562990)		IT-227/82 (AJ880277)		NA-1 (DQ659677)	
**Genotype**	**I**		**II**		**III**		**IV**		**V**		**VI**		**VII**	

	**nt (%)**	**aa (%)**	**nt (%)**	**aa (%)**	**nt (%)**	**aa (%)**	**nt (%)**	**aa (%)**	**nt (%)**	**aa (%)**	**nt (%)**	**aa (%)**	**nt (%)**	**aa (%)**

**NP**	87.5	93.1	84.8	91.8	86.7	92.7	89.2	94.1	89.5	94.1	90.1	95.1	91.8	94.9

**P**	86.4	84.3	88.1	87.1	85.8	84.3	87.5	87.6	86.7	82.3	87.6	82.8	88.3	85.6

**V**	86.9	80.0	91.8	85.5	85.8	81.7	87.8	83.8	85.4	79.2	86.4	77.5	86.1	83.3

**M**	85.1	91.0	84.3	89.3	86.3	91.8	87.7	94.2	88.7	96.7	90.0	96.2	90.9	97.5

**F**	86.5	91.3	84.1	88.8	86.0	91.5	88.5	93.5	88.1	92.8	89.6	95.3	90.9	95.7

**HN^a^**	84.4	88.8	81.2	85.8	84.3	87.8	86.0	89.0	87.8	91.1	88.6	92.5	89.4	93.5

**L**	88.0	94.9	87.0	92.7	87.6	94.3	88.9	95.2	89.8	96.0	90.5	95.6	90.3	95.1

**Complete Genome**	85.2	-	84.0	-	85.0	-	86.7	-	87.4	-	88.3	-	89.0	-

^a^For reliable comparisons, all the HN proteins were cut to generate HN protein of same length (571 aa), as that of Chicken/BYP/Pakistan/2010

#### P gene analysis

The P protein is the only multi-coding protein of NDV, identified so far. The P gene undergo RNA editing due to stuttering of the polymerase complex over the template and leads to insertion of non-templated nucleotide(s) (guanine, G) in the editing site. The editing site (AAAAAGGG) in Chicken/BYP/Pakistan/2010 was found at position 394-401. The P gene transcribe into P protein (unedited), V protein (+1 frame-shift) and W protein (+2 frame-shift). These three proteins share N terminus and have unique C-terminus [4]. The P gene was the most variable gene among the six NDV genes when compared with representatives of each genotype. Therefore, it is believed that the P gene is an evolutionary strategy of the virus that increases the coding capacity of the genome [16]. The lengths of the V proteins of almost all NDV strains including Chicken/BYP/Pakistan/2010 are identical (239 aa). Comparison of the V protein from representative NDVs from each lineage revealed that the C-terminus of the V protein is highly conserved and this starts from the most variable region of editing site [11,17]. The seven cysteine residues, which have been identified in the C-terminus are responsible for the co-ordination of two zinc atoms to form a unique finger fold. These residues were located at positions 196, 200, 212, 214, 217, 221 and 224 in Chicken/BYP/Pakistan/2010. However, the length of W protein is highly variable (147 aa to 227 aa). Our sequencing found a length of 227 aa for the W protein of Chicken/BYP/Pakistan/2010. It has been shown that the length of W protein is not associated with any virulence of NDV [18].

#### M gene analysis

The M protein of NDV is primarily involved in the assembly and intracellular transport of NDV components. Sequencing in our study indicated that the length of the M protein of Chicken/BYP/Pakistan/2010 was 364 aa which is identical to all other NDV strains except Herts/33 with a length of 380 aa. The M protein of NDV is localized in the nucleus and this localization is mediated through nuclear localization signals (NLS). The NLS ^246^DKKGKKVTFDKIEEKIRR^263 ^was identified for Chicken/BYP/Pakistan/2010 as for other strains of NDV.

#### HN gene analysis

**Figure 4:**
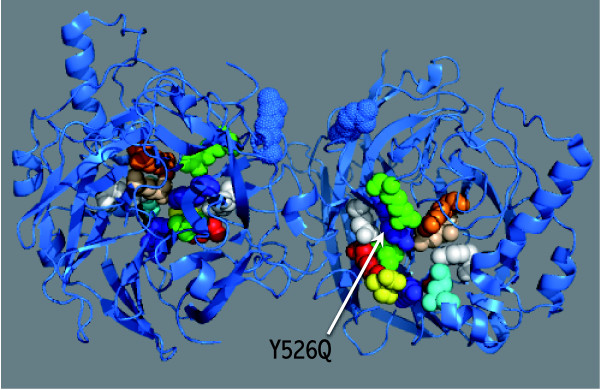
**The crystal structure of HN protein of NDV (PDB ID number **1E8U**)**. The important residues responsible for receptor binding are highlighted. The Y526Q substitution is marked with an arrow. The visualization and annotation were carried out using MacPyMole (version 1.3).

The HN protein of NDV is a multifunctional protein that plays a crucial role in virus infectivity. The primary role of the HN protein is to bind with sialic acid containing receptors, which play key role in the initiation of viral infection in the host. Special emphasis was given to HN protein due to the isolation of Chicken/BYP/Pakistan/2010 from healthy backyard poultry flocks. In order to understand the contribution of HN protein in receptor specificity, the analysis of deduced amino acid sequence of HN protein was performed. Out of 14 residues (174, 175, 198, 236, 258, 299, 317, 401, 416, 498, 516, 526 and 547) identified essential for the receptor recognition, Chicken/BYP/Pakistan/2010 showed a substitution at 526 (Y526Q) (Table 4 and Figure 4). Interestingly, a study conducted by Khattar et al., [19] indicated that the NDV strain Beaudette C if mutated at position 526 (Y526Q) reduced the neuraminidase receptor binding and fusion activities of NDV and thus lead to attenuation of viral virulence in eggs and young birds.

**Table 4:** Homology among all the genotypes in the amino acid residues of HN protein of NDV recognized as important for receptor recognition

Virus	Amino acid residues important for receptor recognition												
	
	174	175	198	236	258	299	317	401	416	498	516	526	547
**Ulster (AY562991)**	R	I	D	K	E	Y	Y	E	R	R	R	Y	E

**LaSota (AY845400)**	.	.	.	.	.	.	.	.	.	.	.	.	.

**Mukteswar (EF201805)**	.	.	.	.	.	.	.	.	.	.	.	.	.

**Hert/33 (AY7411404)**	.	.	.	.	.	.	.	.	.	.	.	.	.

**Largo/71 (AY562990)**	.	.	.	.	.	.	.	.	.	.	.	.	.

**IT-227/82 (AJ880277)**	.	.	.	.	.	.	.	.	.	.	.	.	.

**NA-1 (DQ659677)**	.	.	.	.	.	.	.	.	.	.	.	.	.

**Chicken/BYP/Pakistan/2010**	.	.	.	.	.	.	.	.	.	.	.	Q	.

It has been observed that there are seven antigenic sites within HN protein that are involved in the formation of a continuum in the three dimensional conformation of HN molecules as demonstrated in Table 5. A total of five substitutions, I514V, D569V, N263K, E347D and R353Q have been observed in region 2, 3 and 14 of the HN protein of Chicken/BYP/Pakistan/2010. There have been five conserved potential glycosylation sites identified at position 119 (NNSG), 341 (NNTC), 433 (NKTA), 481 (NHTL) and 538 (NKVY) whereas one site at position 508 was absent in Chicken/BYP/Pakistan/2010 which is considered non-conserved among paramyxoviruses [20].

**Table 5:** Amino acid residues recognized as antigenic sites for HN protein

Virus/Region	Antigenic amino acid residues																		
	
	1	2				3			4			12		14			23		
	**345**	**513**	**514**	**521**	**569**	**263**	**287**	**321**	**332**	**333**	**356**	**494**	**516**	**347**	**350**	**353**	**193**	**194**	**201**

**Ulster (AY562991)**	P	R	I	S	D	N	D	K	G	K	K	G	R	E	Y	R	L	S	H

**LaSota (AY845400)**	.	.	.	.	.	.	.	.	.	.	R	G	.	.	.	.	.	.	.

**Mukteswar (EF201805)**	.	.	.	.	G	K	.	.	.	R	.	.	.	.	.	.	.	.	.

**Hert/33 (AY7411404)**	.	.	.	.	.	S	.	.	.	R	.	.	.	.	.	.	.	.	.

**Largo/71 (AY562990)**	.	.	.	.	N	K	.	R	.	R	.	N	.	.	.	.	.	.	.

**IT-227/82 (AJ880277)**	.	.	V	.	G	K	.	.	.	.	.	.	.	G	.	.	.	.	.

**NA-1 (DQ659677)**	.	.	V	.	.	K	.	.	.	.	.	.	.	.	.	.	.	.	.

**Chicken/BYP/Pakistan/2010**	.	.	V	.	V	K	.	.	.	.	.	.	.	D	.	Q	.	.	.

There have been three lengths reported for the HN protein of NDV, which depends upon genotype. The lengths of HN protein in genotype I is 616 aa, in genotype II is 577 aa, whereas the HN length in genotypes IV, V and VII are 571 aa, irrespective of their pathogenicity. As expected, being a genotype VII, Chicken/BYP/Pakistan/2010 had a HN length of 571 aa.

#### F gene analysis

The F protein is initially synthesized as an inactive precursor (F_0_), which is cleaved by host-cell proteases into F1 and F2, which constitute biologically active proteins, still connected through disulfide-linked chains. The primary function of the F protein is to initiate the fusion of viral surface to that of host cell membrane [3]. As a rule of thumb, a consensus amino acid sequence of ^112^R/K-R-Q-R/K-R-F^117 ^is present in velogenic strains of NDV and ^112 ^G/E-K/R-Q-G/E-R-L^117 ^is present in lentogenic viruses. Despite the fact that Chicken/BYP/Pakistan/2010 was isolated from healthy backyard poultry flocks, it contains ^112^R-R-Q-K-R-F^117^, which corresponds to the cleavage site of velogenic viruses and was in accordance with its MDT and ICPI.

#### L gene analysis

The L protein is the largest protein among the six proteins of NDV and function as a RNA-dependent-RNA polymerase. A motif (GDNQ) is considered responsible for the polymerase activity and is highly conserved within L proteins of non-segmented, negative-sense RNA viruses. In Chicken/BYP/Pakistan/2010, a corresponding sequence was found (GDNQ) at position 750-753 aa. In comparison with representatives of each genotype, the L protein of Chicken/BYP/Pakistan/2010 was found to be the most conserved protein among the six proteins of NDV.

## Discussion

This study presents the first characterization of an NDV isolated from rural poultry in Pakistan, and can serve as a basis for future vaccine design. The study highlights the importance of rural poultry in the epizootiology of the disease in the country. According to the criteria set by the OIE, the virulence of NDV is associated with ICPI and the cleavage site in the F protein [12,21]. ICPI of 0.7 or greater in day-old chicken or presence of three basic amino acids (R or K) at the F protein cleavage sits between residues 113 and 116 indicate the virulent form of NDV. The isolate in current study surprisingly exhibited MDT of 49.6 h in embryonated chicken eggs and ICPI value of 1.5. Moreover, the Chicken/BYP/Pakistan/2010 possessed 112R-R-Q-K-R-F117 at the cleavage site, the same as of the velogenic strains of NDV [5]. All these facts indicate that the rural poultry can harbour virulent strains of NDV without showing clinical signs, and that it consequently may act as silent carriers and constitute a potential threat to the commercial poultry. On the other hand, this finding also indicates that local breeds are more resistant and can sustain the virulent form of the disease. However, the presence of other secondary infections (avian influenza along with other viral, bacterial or parasitic infections) and immuno-compromising husbandry factors may provoke the disease, which warrant further studies in the rural poultry chickens. Beside these facts, sample collection during incubation period of the disease, can't be ruled out in this study. Currently, there is great wealth of reports discussing the error prone genome transcription during replication of RNA viruses such as NDV. This process helps viruses escape host immune defences, alter pathogenicity and host range, and evade diagnostic tests. To properly understand these mechanisms, it is of paramount importance to fully characterize the viruses in order to study within-host dynamics and genetic variation, relate dynamics and variation to transmission, and to reconstruct transmission trees at high resolution.

It has been demonstrated that virulence of NDV is a multigenic trait as that of influenza viruses which is mainly contributed by HN, V and L proteins of NDV [22-24]. The essential role of L protein in pathogenicity can be shown by e.g. the Beaudette C (BC) strain of NDV, which if it carries the L protein of LaSota increases its virulence. Recombinant viruses deficient in V protein have a tendency to be attenuated and show impaired growth in cell culture, an indication of V protein involvement in the virulence of the virus by down regulation of the host immune response [24]. Applying reverse genetics, it has been concluded that BC strains show decreased virulence when HN protein was replaced with LaSota, and that the virulence of LaSota strain was increased when HN protein was replace with HN protein of BC. Moreover, the role of 5'UTR of the HN gene has been shown to be essential for the pathogenicity of the virus. The HN protein of Chicken/BYP/Pakistan/2010 showed an Y526Q substitution at the receptor-binding site. This site has been demonstrated essential for the neuraminidase receptor binding, and fusion activities of the NDV [19]. This substitution leads to reduction in the pathogenicity of NDV both *in vitro *and *in vivo*. This fact might explain the reduced or attenuated pathogenicity of the Chicken/BYP/Pakistan/2010 in rural poultry birds. However, it is to mention that the isolate regained the pathogenicity when chicken embryos were infected and this may demand further studies to look for this mechanism at molecular level.

The isolate under study was classified in genotype VII, which is the pre-dominant genotype responsible for ND outbreaks in the Asian countries, including Pakistan. However, a previous study conducted on NDV isolates from Karachi showed that at least two different genotypes (VI and VII) are present in the country [13]. All these isolates were clustered with isolates from China and India suggesting they were derived from common sources and spread between the countries and the role of wild bird population cannot be ignored. It has been reported before that genetically distinct genotypes may co-circulate in a region and cause disease [25]. Genotype VII is predominantly reported from Asian countries such as Korea, Taiwan and China since 1980. The F gene based analysis suggested that Chicken/BYP/Pakistan/2010 is closest to the Chinese NDV isolates among bordering countries. It can be mentioned that the H5N1 highly pathogenic influenza viruses in Pakistan are also genetically related to isolates from China [15,26]. There could be several possibilities, based on ground realities, for this fact. The movement of contaminated material and illegal infected material across the border may be the main factor in this transmission. The wild birds may also play role in dissemination of these viruses [27]. Therefore, the extensive surveillance of the wild birds for the NDV is a fundamental requirement for understanding the epidemiology of this virus as it has usually been practiced for avian influenza viruses.

Several form of vaccines carrying different strains of NDV (F, LaSota and Mukteswar) have been used in Pakistan [28]. The complete genome sequence comparison to the representative genome of each genotype revealed that Chicken/BYP/Pakistan/2010 showed least identity to the LaSota (AY845400) and Mukteswar (EF201805) strain with percentage identity of 84.0 and 85.0, respectively. Being grouped in lineage VII, NA-1 (DQ659677) showed highest identity to Chicken/BYP/Pakistan/2010. Among all the complete genomes available in the GenBank, Chicken/BYP/Pakistan/2010 was highly identical to that of Sterna/Astr/2755/2001 (GenBank accession number AY865652). This isolate is identified from little Tern (*Sterna albifrons Pallas*) in the Volga river delta in Russia. Considering the transboundary nature of the disease, Chicken/BYP/Pakistan/2010 showed 85.2% and 84.3% sequence identity to two Indian isolates, NDV-4 (HM357251) and NDV2K17 (HQ902590), respectively. To our knowledge, there is no complete genome sequence of NDV available from other countries in the region such as Iran, Afghanistan, Bangladesh and Nepal.

In spite of extensive potential of sequence data evaluation in epizootiological investigations, the information on the geographical distribution of epizootic NDV genotypes is extremely limited and mainly insufficient for epidemiological investigations, especially in the South-East Asian countries. Moreover, to establish epidemiological link and to understand introduction of ND to a new location (as of European NDV strains-genotype VII), it is fundamental requirement to characterize the viruses in the regions where this data is lacking. It is even more important in country like Pakistan being major exporter of wild birds to the EU, sending tens of thousands of parrots and other birds destined for European pet markets each year, particularly to Italy, Spain, Portugal, and Greece [29]. Recently, it has been observed that the parrots, lovebirds and finches imported from Pakistan to Italy were carrying exotic Newcastle disease [29]. Such facts impose national collections of NDV strains, which would prove useful in future epidemiological investigations.

To conclude, we have characterized a full-length genome of a velogenic NDV from healthy backyard poultry flock, which belongs to the genotype VII along with other Asian isolates. We have provided evidence for the existence of novel genetic group of NDV in Pakistan and that substantial changes in the probe site of M-gene based real-time PCR. This isolate will be valuable in analyzing the genetic nature of APMV-1 not only in Pakistan, but also in other neighboring countries. Findings in this study advance the currently available full genome data on APMV-1. Furthermore, we have established a domestic reference virus for future studies which would lead to development and selection of appropriate strain of NDV for vaccine.

## Methods

### Virus collection and isolation

In an attempt to screen for pathogens, cloacal swabs, tracheal swabs and blood samples were collected from apparently healthy poultry flocks kept at homes of farm-assistants working in nearby commercial poultry farms. Samples from individual birds were inoculated in five 10-day-old specific pathogen free chicken embryos via allantoic cavity. Three days after inoculation, the allantoic fluid was harvested and clarified by centrifugation at 4000 × g for 30 min at 4°C. The supernatant was collected and used to run for standard hemagglutination inhibition test using specific antisera to the reference strains of NDV (avian paramyxovirus type I). Samples showing high HA titer were divided into working stocks and stored at -20°C. These allantoic fluids were used in pathogenicity assessment and for sequence analysis. For genome detection and characterization, allantoic fluid from each flock was stored on QIAcard FTA Indicator Four Spots (Qiagen, Hilden, Germany), which preserve nucleic acids and inactivate the virus. The samples were shipped at ambient temperature from Pakistan to the Swedish University of Agricultural Sciences, Uppsala, Sweden, for processing.

### Intracerebral pathogenicity index (ICPI)

ICPI was performed in ten 1-day old chicks by inoculation of 50 μl of allantoic fluid with hemagglutination (HA) titer of more than 2^4 ^and diluted 10 fold in PBS without antibiotics, as recommended [21]. The birds were kept under observation for one week and examined after each 24 h. The birds scored an ICPI of 0.7 were declared as lentogenic strain of NDV and the birds with ICPI of more than 1.5 were considered velogenic. The NDV strains with intermediate ICPI values were designated as mesogenic.

### Mean death time (MDT) calculation

For MDT, allantoic fluid carrying virus was 10-fold diluted in PBS (pH 7.2) for embryonated chicken eggs inoculations. The MDT induced by minimal lethal dose was determined by the procedure described before [21]. The isolates with MDT of up to 60 h, from 61 to 90 h and more than 90 h were designated as velogenic, mesogenic and lentogenic, respectively.

### RNA elution from Qiacard FTA indicator

The RNA was eluted from Qiacard FTA Indicator (Qiagen) impregnated with allantoic fluid as recommended by the manufacturer (preparation of isolated RNA from FTA_Cards, Rev 1 10/17/07; Whatman, Hilden, Germany) with following modifications. Using a 2.0-mm-diameter Harris micropunch (Whatman), one punch for each sample was removed according to manufacturer's protocol (BD09; Whatman) and placed in separate 1.5 ml microfuge tubes; 200 μl Tris-EDTA buffer (10 mm Tris-HCl, pH 8.0, 0.1 mm EDTA) was used instead of RNA processing buffer and incubated for 15 min (flicking tubes 3 times over the course of incubation) on ice.

### F and M gene based real-time PCR screening

The detection of nucleic acid for NDV in the Qiacard FTA Indicator was performed using real-time PCR for M and F genes, as previously described [30,31]. The reaction was carried out in a Rotor-Gene 6000 real-time analyzer (Qiagen).

### Genome amplification and sequencing

For the amplification of complete genome of NDV, a set of 22 primer pairs were designed using the consensus of 52 full-length APMV-1 sequences available in the GenBank (Additional file 1:Table S1). These degenerate primers of 16-20 nucleotides in length with similar melting temperatures were used for both PCR amplification and sequencing of each gene of NDV. 3 μl of eluted RNA extract (the same as used in real-time PCRs) was supplemented in a 25 μl reaction of One-Step RT-PCR kit (Qiagen) for amplification of each fragment. The amplified PCR products were gel extracted and processed for sequencing using ABI PRISM BigDye Terminator version 3.1 (Applied Biosystems), according to the manufacturer's instructions. Sequences were analysed with an automated nucleic acid analyzer (ABI PRISM 3100; Applied Biosystems). Each DNA fragment was sequenced at least twice in both directions.

### Sequence and phylogenetic analysis

Sequence assembly and editing were performed using the SEQMAN program from DNASTAR Lasergene suite 9 (version 9.0.4 39; DNASTAR, Inc., Madison, WI, USA). To determine the phylogenetic relationships between APMV-1 viruses previously characterized from Asia and other parts of the world, the sequences of the complete open reading frame of the F and HN genes were compared to the corresponding region of representative viruses available in GenBank (http://www.ncbi.nlm.nih.gov/), for which the genotype was known. All the sequences were aligned in BioEdit (version 7.0.8) using clustalW method and were cut to equal length. The sequences were then used to construct phylogenetic tree by Bayesian methods available in the computer program MrBayes version 3.1.2 [32] (parameter values available from authors on request). Nucleotide similarity was calculated using the MegAlign programme in DNASTAR Lasergene suite 9 (version 9.0.4 39; DNASTAR, Inc., Madison, WI, USA). Mean distances within genotype VII were calculated using PASC (PAirwise Sequence Comparisons) in MEGA4 software.

### Visualization and annotation of the HN protein

The crystal structure of HN protein of NDV was downloaded from PBD under PDB ID number 1E8U as described before [20]. All the annotation for receptor binding sites and visualization was carried out using MacPyMole (version 1.3).

### Accession number

The complete genome sequence was submitted to GenBank with the accession number JN682210.

## Competing interests

The authors declare that they have no competing interests.

## Authors' contributions

Conceived and designed the experiments: MM, SZ, MB. Performed the experiments: MM, MA, MTK. Analyzed the data: MM, MB. Wrote the paper: MM, SZ, MB. All the authors have read and approved the final manuscript.

## Supplementary Material

Additional file 1 **Table S1**. Primers for whole genome amplification and sequencing. Click here for file

## References

[B1] de Leeuw O Peeters B Complete nucleotide sequence of Newcastle disease virus: evidence for the existence of a new genus within the subfamily Paramyxovirinae J Gen Virol 1999 80 Pt 1 131 136 9934695 10.1099/0022-1317-80-1-131

[B2] Kolakofsky D Roux L Garcin D Ruigrok RW Paramyxovirus mRNA editing, the "rule of six" and error catastrophe: a hypothesis J Gen Virol 2005 86 1869 1877 10.1099/vir.0.80986-0 15958664

[B3] Lamb RA Kolakofsky D Paramyxoviridae: The Viruses and Their Replication 2001 4 Hagerstown: Lippincott Williams & Wilkins

[B4] Steward M Vipond IB Millar NS Emmerson PT RNA editing in Newcastle disease virus J Gen Virol 1993 74 Pt 12 2539 2547 8277263 10.1099/0022-1317-74-12-2539

[B5] Collins MS Bashiruddin JB Alexander DJ Deduced amino acid sequences at the fusion protein cleavage site of Newcastle disease viruses showing variation in antigenicity and pathogenicity Arch Virol 1993 128 363 370 10.1007/BF01309446 8435046

[B6] Lomniczi B Wehmann E Herczeg J Ballagi-Pordany A Kaleta EF Werner O Meulemans G Jorgensen PH Mante AP Gielkens AL Newcastle disease outbreaks in recent years in western Europe were caused by an old (VI) and a novel genotype (VII) Arch Virol 1998 143 49 64 10.1007/s007050050267 9505965

[B7] Herczeg J Wehmann E Bragg RR Travassos Dias PM Hadjiev G Werner O Lomniczi B Two novel genetic groups (VIIb and VIII) responsible for recent Newcastle disease outbreaks in Southern Africa, one (VIIb) of which reached Southern Europe Arch Virol 1999 144 2087 2099 10.1007/s007050050624 10603164

[B8] Aldous EW Mynn JK Banks J Alexander DJ A molecular epidemiological study of avian paramyxovirus type 1 (Newcastle disease virus) isolates by phylogenetic analysis of a partial nucleotide sequence of the fusion protein gene Avian Pathol 2003 32 239 256 10.1080/030794503100009783 12850913

[B9] Huang Z Panda A Elankumaran S Govindarajan D Rockemann DD Samal SK The hemagglutinin-neuraminidase protein of Newcastle disease virus determines tropism and virulence J Virol 2004 78 4176 4184 10.1128/JVI.78.8.4176-4184.2004 15047833 PMC374304

[B10] Czegledi A Ujvari D Somogyi E Wehmann E Werner O Lomniczi B Third genome size category of avian paramyxovirus serotype 1 (Newcastle disease virus) and evolutionary implications Virus Res 2006 120 36 48 10.1016/j.virusres.2005.11.009 16766077

[B11] Munir M Linde AM Zohari S Stahl K Baule C Engstrom B MLH Renström Berg M Whole genome sequencing and characterization of a virulent Newcastle disease virus isolated from an outbreak in Sweden Virus Genes 2011 43 261 271 10.1007/s11262-011-0636-2 21667282

[B12] Truszczynski M, et al OIE, in Newcastle Disease International Animal Health Code--Mammals, Birds and Bees 1992 6 Epizooties. OIE, Paris 157 165

[B13] Khan TA Rue CA Rehmani SF Ahmed A Wasilenko JL Miller PJ Afonso CL Phylogenetic and biological characterization of Newcastle disease virus isolates from Pakistan J Clin Microbiol 2010 48 1892 1894 10.1128/JCM.00148-10 20237105 PMC2863937

[B14] Sakaguchi T Toyoda T Gotoh B Inocencio NM Kuma K Miyata T Nagai Y Newcastle disease virus evolution. I. Multiple lineages defined by sequence variability of the hemagglutinin-neuraminidase gene Virology 1989 169 260 272 10.1016/0042-6822(89)90151-7 2705297

[B15] Fiebig L Soyka J Buda S Buchholz U Dehnert M Haas W Avian influenza A(H5N1) in humans: new insights from a line list of World Health Organization confirmed cases, September 2006 to August 2010 Eurosurveillance 2011 16 pii = 9941 21871222

[B16] Jordan IK Sutter BAt McClure MA Molecular evolution of the Paramyxoviridae and Rhabdoviridae multiple-protein-encoding P gene Mol Biol Evol 2000 17 75 86 10666708 10.1093/oxfordjournals.molbev.a026240

[B17] Linde AM Munir M Zohari S Stahl K Baule C Renstrom L Berg M Complete genome characterisation of a Newcastle disease virus isolated during an outbreak in Sweden in 1997 Virus Genes 2010 41 165 173 10.1007/s11262-010-0498-z 20640497

[B18] Wei D Yang B Li YL Xue CF Chen ZN Bian H Characterization of the genome sequence of an oncolytic Newcastle disease virus strain Italien Virus Res 2008 135 312 319 10.1016/j.virusres.2008.03.003 18420299

[B19] Khattar SK Yan Y Panda A Collins PL Samal SK A Y526Q mutation in the Newcastle disease virus HN protein reduces its functional activities and attenuates virus replication and pathogenicity J Virol 2009 83 7779 7782 10.1128/JVI.00536-09 19474107 PMC2708642

[B20] Crennell S Takimoto T Portner A Taylor G Crystal structure of the multifunctional paramyxovirus hemagglutinin-neuraminidase Nat Struct Biol 2000 7 1068 1074 10.1038/81002 11062565

[B21] Alexander DJ Newcastle disease. Diseases of poultry 2003 11 Ames: Iowa State University Press

[B22] Yan Y Rout SN Kim SH Samal SK Role of untranslated regions of the hemagglutinin-neuraminidase gene in replication and pathogenicity of newcastle disease virus J Virol 2009 83 5943 5946 10.1128/JVI.00188-09 19321607 PMC2681929

[B23] Peeters BP Gruijthuijsen YK de Leeuw OS Gielkens AL Genome replication of Newcastle disease virus: involvement of the rule-of-six Arch Virol 2000 145 1829 1845 10.1007/s007050070059 11043944

[B24] Huang Z Krishnamurthy S Panda A Samal SK Newcastle disease virus V protein is associated with viral pathogenesis and functions as an alpha interferon antagonist J Virol 2003 77 8676 8685 10.1128/JVI.77.16.8676-8685.2003 12885886 PMC167241

[B25] Mase M Inoue T Imada T Genotyping of Newcastle disease viruses isolated from 2001 to 2007 in Japan J Vet Med Sci 2009 71 1101 1104 10.1292/jvms.71.1101 19721367

[B26] Uyeki TM Human infection with highly pathogenic avian influenza A (H5N1) virus: review of clinical issues Clin Infectious Diseases Official Publ Infectious Diseases Soc Am 2009 49 279 290 10.1086/600035 19522652

[B27] Munir M Linde AM Zohari S Stahl K Baule C Holm K Engstrom B Berg M Complete genome analysis of an avian paramyxovirus type 1 strain isolated in 1994 from an asymptomatic black-headed gull (Larus ridibundus) in southern Sweden Avian Dis 2010 54 923 930 10.1637/9086-092409-RESNOTE.1 20608541

[B28] Rehmani SF Newcastle disease vaccination: A comparison of vaccines and routes of administration in Pakistan Preventive Veterinary Med 1996 25 241 248 10.1016/0167-5877(95)00487-4

[B29] Trust WP Deadly Newcastle disease discovered in parrots and other birds imported from Pakistan to Italy www.parrots.org/pdfs/.../newcastle_disease.pdf 2011. Accessed on 10 Feb, 2011

[B30] Pederson JC National Veterinary Services Laboratories testing protocol real-time RT-PCR for detection of exotic Newcastle disease virus in clinical samples USDA APHIS, ed. AVPRO1505.03 2005 U.S. Department of Agriculture, APHIS, NVSL, Ames, IA.

[B31] Wise MG Suarez DL Seal BS Pedersen JC Senne DA King DJ Kapczynski DR Spackman E Development of a real-time reverse-transcription PCR for detection of newcastle disease virus RNA in clinical samples J Clin Microbiol 2004 42 329 338 10.1128/JCM.42.1.329-338.2004 14715773 PMC321685

[B32] Ronquist F Huelsenbeck JP MrBayes 3: Bayesian phylogenetic inference under mixed models Bioinformatics 2003 19 1572 1574 10.1093/bioinformatics/btg180 12912839

