# Supplementary material for: Genomic and biological characterization of a velogenic Newcastle disease virus isolated from a healthy backyard poultry flock in 2010
Source: Virol J. 2012 Feb 16;9:46. doi: 10.1186/1743-422X-9-46 (PMC3295720; doi:10.1186/1743-422X-9-46)
Supplement: Additional file 1 — Table S1. Primers for whole genome amplification and sequencing. [file 1743-422X-9-46-S1.DOC]

Table 1S. Features of the primers used in this study.

| Primer name | Sequence (5′ to 3′) | Position^c^ |
| --- | --- | --- |
| 1-S^a^ | ACCAAACAGAGAATCCGT | 1-18 |
| 1-A^b^ | GTTAGCGARGATGCCAA | 315-299 |
| 2-S | GTCCCGGTATTCACYCT | 224-240 |
| 2-A | ATGAAGCAGCTCATGCG | 1017-1001 |
| 3-S | GGAYGTAGACTCATACATC | 880-898 |
| 3-A | AAGTACGGGTAGAAGAGR | 1817-1800 |
| 4-S | GAYAACGACACCGACTG | 1565-1581 |
| 4-A | CACAGCATATCATGGACAA | 2444-2426 |
| 5-S | ACCGGAGCAAGCAACTC | 2217-2233 |
| 5-A | CGCCTTGCTCTRAATGGC | 3071-3054 |
| 6-S | GACACTGTCCGTGCATTG | 2943-2900 |
| 6-A | AATCTTGCGCTCAATGTCAC | 3915-3896 |
| 7-S | GTGAAYTTTGTCTCCTTGAC | 3806-3825 |
| 7-A | GAGGCATGTGCRAAAGC | 4779-4763 |
| 8-S | TTGAYGGCAGGCCTCTTG | 4638-4655 |
| 8-A | GTGATAGAAGARCTTGACACCTC | 5550-5528 |
| 9-S | ATAATATGCGTGCCACCTA | 5430-5452 |
| 9-A | ATAYACGGGTAGAACGGT | 6334-6317 |
| 10-S | TGGCTTGGGAAYAATACCCT | 6155-6174 |
| 10-A | TGCAGTGTGAGTGCAACT | 7140-7123 |
| 11-S | CGGACATCTGCAACAGG | 7045-7061 |
| 11-A | GCAGCATACACAACATC | 7997-7981 |
| 12-S | TTCGGGACRATGCTTGAT | 7873-7890 |
| 12-A | GCATTCTGGTTTCACTCAA | 8882-8864 |
| 13-S | TCCAAATTCACAACACRAG | 8730-8748 |
| 13-A | CTTCAAGGGAACAATCATC | 9583-9565 |
| 14-S | AATCAAGCAGCTGAGATG | 9422-9439 |
| 14-A | TGATGGGCCTACCTCAYT | 10388-10371 |
| 15-S | AGAAACGTATCACTGACTG | 10203-10221 |
| 15-A | CAATACTCAAGGCTCTACAC | 11163-11144 |
| 16-S | ATGAGTTGYGTGCAGACAT | 10997-11015 |
| 16-A | TGTGACAGCGGAGATGA | 11874-11858 |
| 17-S | CGGAATAGAAGCTGGTCACC | 11741-11760 |
| 17-A | GCGAGACTTGACTTAGCTATC | 12568-12548 |
| 18-S | GAAGAAGGRGTCAAAGAGG | 12287-12305 |
| 18-A | TGCTACAACAAGAGAAATCC | 13190-13171 |
| 19-S | GACGGRTCACACCAACTT | 12971-12988 |
| 19-A | GGAGCCATCATGAGTCTTCT | 13743-13724 |
| 20-S | TCYTCTTGGTATAAGGCATC | 13634-13653 |
| 20-A | ATGGAAGCTGAGGGTGA | 14616-14600 |
| 21-S | GTCCGTCCATTCTGTGC | 14492-14508 |
| 21-A | TACTTGACTCGTGCTCAACA | 14931-14912 |
| 22-S | CATCACTTAAACAGTGCAC | 14679-14697 |
| 22-A | CACCAAATCTTTGTTTGGT | 15186-15168 |

^a^S: the sense primer, ^b^A: the anti-sense primer.

^c^Positions are based on the full-length sequence of LaSota strain of NDV (GenBank accession number: AF077761). The degenerate bases, e.g., Y 5 T/C, R 5 A/G, are according to the International Union of Pure and Applied Chemistry.
